# Prediction of anti-microtubular target proteins of tubulins and their interacting proteins using Gene Ontology tools

**DOI:** 10.1186/s43141-023-00531-8

**Published:** 2023-07-19

**Authors:** Polani B. Ramesh Babu

**Affiliations:** Center for Materials Engineering and Regenerative Medicine, Bharath Institute of Higher Education and Research, Bharath Institute of Science and Technology, Selaiyur, Tambaram, Chennai India

**Keywords:** Tubulins, Microtubules, Gene Ontology, Protein–protein interactions, Drug targets

## Abstract

**Background:**

Tubulins are highly conserved globular proteins involved in stabilization of cellular cytoskeletal microtubules during cell cycle. Different isoforms of tubulins are differentially expressed in various cell types, and their protein–protein interactions (PPIs) analysis will help in identifying the anti-microtubular drug targets for cancer and neurological disorders. Numerous web-based PPIs analysis methods are recently being used, and in this paper, I used Gene Ontology (GO) tools, e.g., Stringbase, ProteomeHD, GeneMANIA, and ShinyGO, to identify anti-microtubular target proteins by selecting strongly interacting proteins of tubulins.

**Results:**

I used 6 different human tubulin isoforms (two from each of *α*-, *β*-, and *γ*-tubulin) and found several thousands of node-to-node protein interactions (highest 4956 in GeneMANIA) and selected top 10 strongly interacting node-to-node interactions with highest score, which included 7 tubulin family protein and 6 non-tubulin family proteins (total 13). Functional enrichment analysis indicated a significant role of these 13 proteins in nucleation, polymerization or depolymerization of microtubules, membrane tethering and docking, dorsal root ganglion development, mitotic cycle, and cytoskeletal organization. I found *γ*-tubulins (TUBG1, TUBGCP4, and TUBBGCP6) were known to contribute majorly for tubulin-associated functions followed by *α*-tubulin (TUBA1A) and *β*-tubulins (TUBB AND TUBB3). In PPI results, I found several non-tubular proteins interacting with tubulins, and six of them (HTT, DPYSL2, SKI, UNC5C, NINL, and DDX41) were found closely associated with their functions.

**Conclusions:**

Increasing number of regulatory proteins and subpopulation of tubulin proteins are being reported with poor understanding in their association with microtubule assembly and disassembly. The functional enrichment analysis of tubulin isoforms using recent GO tools resulted in identification of *γ*-tubulins playing a key role in microtubule functions and observed non-tubulin family of proteins HTT, DPYSL2, SKI, UNC5C, NINL, and DDX41 strongly interacting functional proteins of tubulins. The present study yields a promising model system using GO tools to narrow down tubulin-associated proteins as a drug target in cancer, Alzheimer’s, neurological disorders, etc.

## Background

Tubulin isoforms play a major role in modulating microtubule structure, dynamics, and mechanics. Their polymerization into microtubules is indispensable for cell division, growth, intracellular trafficking, and cell signaling. It has three functional domains, namely guanine triphosphate (GTP) binding, drug binding, and motor microtubule-associated protein (MAP)-binding domains. Gene mutations of tubulins are associated with a large spectrum of diseases such as cancer, Alzheimer’s, abnormal neuronal migration, neuronal motor impairments, intellectual disability, and epilepsy [1–4]. In eukaryotes, tubulin superfamily includes six types: alpha, beta, gamma, delta, epsilon, and zeta [5, 6]. Human microtubules constitute mixed combinations of these isotypes encoded by different genes on distinct chromosomes. These isotypes differ from one another by divergent sequences at their carboxy-terminal (C-terminal) tail [7–9].

Studies in protein–protein interactions and cellular functions with tubulins help in identifying drug targets and in understanding disease pathologies associated with tubulins. In vitro studies using plant alkaloids demonstrated important ligand-binding domains in tubulin isoforms. The natural and synthetic agents of plant alkaloids such as colchicine, paclitaxel, and vinblastine were demonstrated to interact with tubulin as anti-microtubular drugs [10, 11]. The interaction of anti-microtubular drugs paclitaxel and vinblastine was earlier demonstrated as anticancer drugs in aggressive metastatic tumors. The functional roll of *β*-III tubulin in voltage-dependent anion channel (VDAC) was determined using brain synaptosomes [12]. Cell signaling studies of tubulin in antigen-mediated mast cells suggested new strategies in the treatment of allergies and inflammatory diseases. The overexpression of *β*-II tubulin was reported to promote cancer growth and metastasis and observed as a useful prognostic marker. The inhibition of proteins with anti-apoptotic pathway plays a major role in understanding the development of anti-microtubular drugs for anticancer drug discovery [13]. The overexpression of BCL2, an apoptosis regulatory protein, was earlier shown to sensitize tumor cells to programmed cell death induced by anti-microtubule drugs. Various isoforms of tubulin were identified as prognostic markers in solid tumors [14], for example, *β*-III tubulin has reportedly overexpressed in rectal cancer, gastric cancer, and gliomas.

Multiple isoforms of tubulin are reported to maintain the stability of microtubules named as microtubular associated proteins (MAPs) [15]. However, their combined functional role is incompletely understood. Based on the curated dataset using sequences from 611 proteins, an online computational tool, namely MAP analyzer, was designed as MAP predictor to identify microtubule-interacting proteins based on their sequence motifs. This tool contains four types of microtubule-related proteins: (1) proteins directly binding to tubulins, (2) proteins altering the microtubule organization and dynamics, (3) proteins colocalizing with microtubules, (4) and indirectly interacting microtubule proteins. This database can be used with protein IDs and protein names obtainable through UniProtKB, RefSeq, etc. Most of the MAPs are reported to interact with microtubule by binding with polymerized or depolymerized tubulin dimers for stabilizing the microtubules. Several MAPs are used as drug targets and MAP inhibitors to be used in clinical trials [16, 17]. Promising approach to find a suitable microtubule drug target depends on understanding tubulin molecular signaling pathways and their protein–protein interactions, which can be performed using GO tools. In the present study, I wanted to identify druggable target proteins of tubulin and their associated proteins by identifying strongly interacting proteins of *α*-, *β*-, and *γ*-tubulins using web-based GO tools.

## Methods

### Selection of tubulin isoforms for PPI analysis

**Fig. 1:**
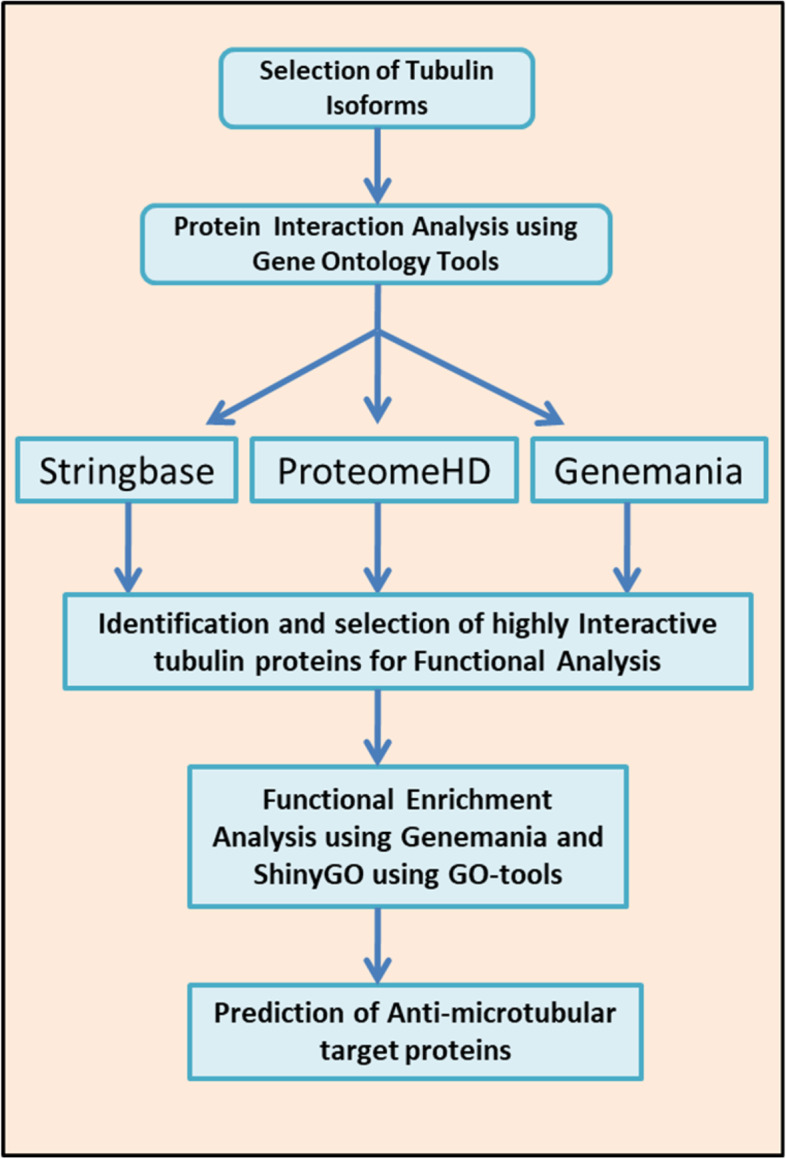
Flow chart depicting the steps involved in identification of tubulin interacting proteins and functional enrichment methods

In the present study, I used six tubulin isoforms (6TI), two from each of human *α*-, *β*-, and *γ*-tubulin, e.g., TUBA1A, TUBA1B, TUBB, TUBB3, TUBG1, AND TUBGCP2, respectively, for PPI analysis. Our working model involved selection of strongly interacting proteins of tubulin superfamily using open-source web-based GO tools (Fig. 1). In Stringbase cutoff confidence score of 0.44, GeneMANIA as per version 3.6.0 default setup and ProteomHD cutoff score of 0.9 were used for PPI search. Our selection process for identifying final set of strongly interacting protein of tubulin for functional analysis involved three steps. The first step involved use of all 6TI together in Stringbase, GeneMANIA, and ProteomeHD, second step involved use of each isoform separately, and final step involved use of all the pooled dataset in GeneMANIA.

### PPI analysis using String database

Stringbase version 11.5 is an open-source web-based database for PPI analysis available at www.string-db.org [18, 19]. It depicts functional association between two proteins jointly contributing to a specific function. The interaction between two proteins does not need physical interaction; it would be sufficient if some part of overlapping occurred functionally in a pathway or functional map. By this definition, proteins which are found inhibitory in action can be functionally associated in the pathway. In this PPI search, the data were derived through various modes from curated databases, experimentally determined, gene neighborhood, gene fusions, co-occurrences, co-expressions, and protein homology. In PPI analysis, the edges are representation between two interacting nodal proteins as predicted edges derived based on experimental evidences and protein associations from 12 different biological data sources. The obtained data from PPI analysis were saved into Excel sheet by downloading tabular text output, and top-level interacting proteins were selected based on highest score values.

### PPI analysis using ProteomeHD

ProteomeHD is an open-source functional annotation tool designed based on R script which builds a database of co-regulated proteins using unsupervised machine learning. This PPI analysis tool is available as an interactive and functionally annotated map at www.proteomeHD.net [20]. The results were obtained with dual cellular functions which are more informative than mRNA co-expression analysis. It provides functional insights that are difficult to obtain by other proteomics approaches. The results of proteins with co-regulation score cutoff value was set at 0.9, and interaction data was obtained in CSV format to identify top-level interacting proteins based on highest score values.

### PPI analysis using GeneMANIA

GeneMANIA (version 3.6.0) is an open-source protein network search engine available freely at www.genemania.org [21]. It finds new members in gene network and builds weighted functional interaction data for each gene based on their predictive value for the query list. It can often find additional genes from the network giving more weightage to physical interaction or predicted physical interactions and prioritize genes for functional assays. It builds network based on millions of interactions obtained from IRefIndex, GEO, BioGRID, and I2D as well as from organism-specific functional genomics data sets. The resulting functional and interaction data can be downloaded into Excel sheet through notepad for identifying top-level interacting genes based on highest score values.

### Functional enrichment analysis

At first step, I used 6TI and selected set of strongly interacting proteins of tubulin for functional analysis in GeneMANIA. Wherein, functional categorization of tested gene set with specific FDR value (false discovery rate) with responsible number of genes involved in the network and from the genome were represented in table. The highest number of genes involved were shown first. Next, I used open-source web-based GO tool ShinyGO version 0.76 for high-level functional enrichment analysis at http://bioinformatics.sdstate.edu/go/ [22] which produces hierarchical clustering trees, networks summarizing overlapping terms/pathways, protein–protein interaction networks, gene characteristics plots, and enriched promoter motifs based on annotation from Ensembl. The number of folds of functional enrichment with number of genes involved with FDR values is graphically represented. Furthermore, the categorization of numbers and groups of genes involved in high-level functional enrichment is represented in tabular format downloadable directly from the database. The correlation of significant functional pathway enrichment is represented through hierarchical tree clustering, and results were downloaded in PNG format from database.

## Results

### Selection of strongly interacting proteins of tubulins

**Fig. 2:**
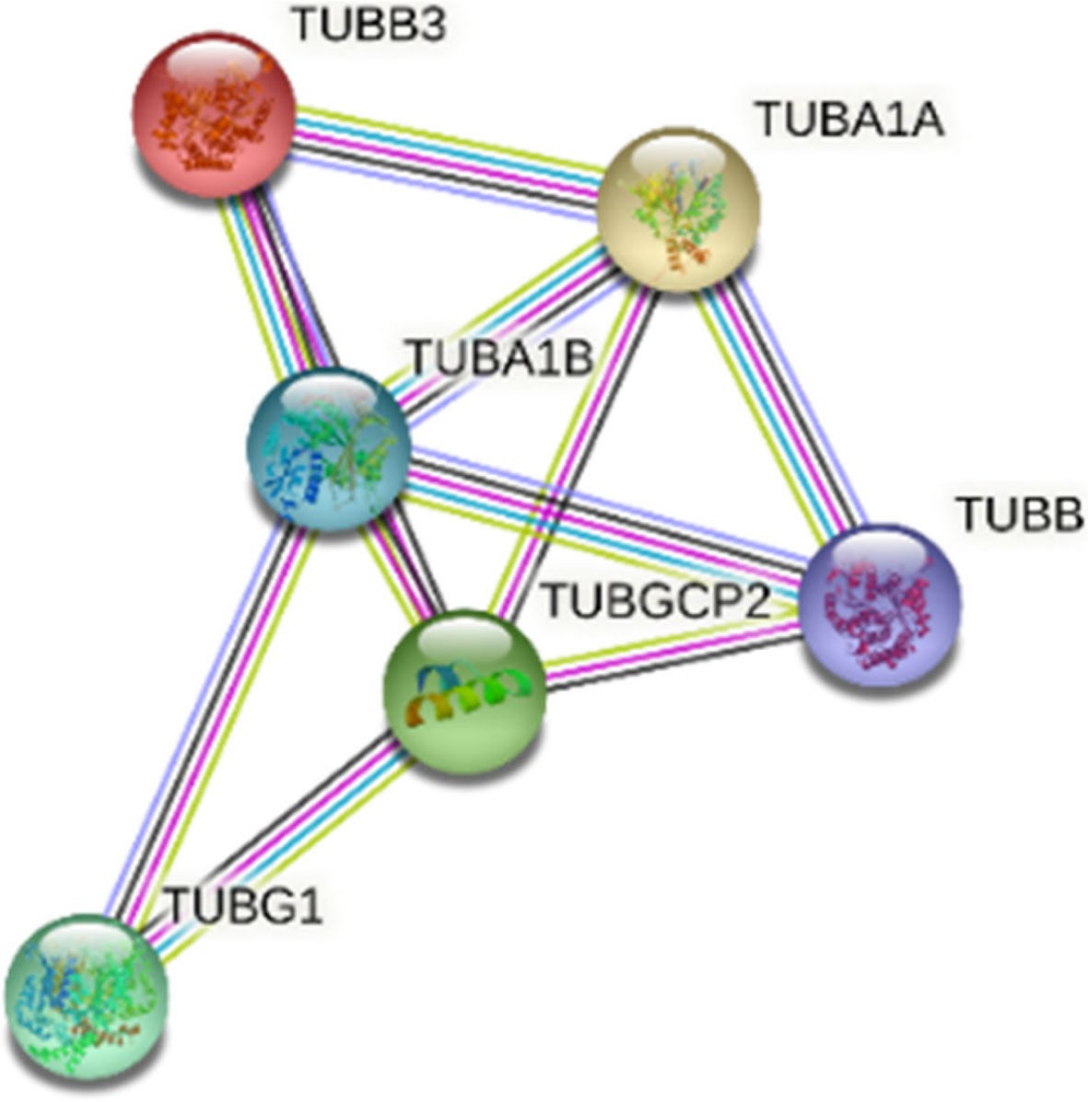
Six selected tubulin isoforms PPI in Stringbase tool. Network nodes details are as follows: number of nodes: 6; number of edges 11; avg. local clustering coefficient 0.839; PPI enrichment *p*-value 1.54e-14

**Fig. 3:**
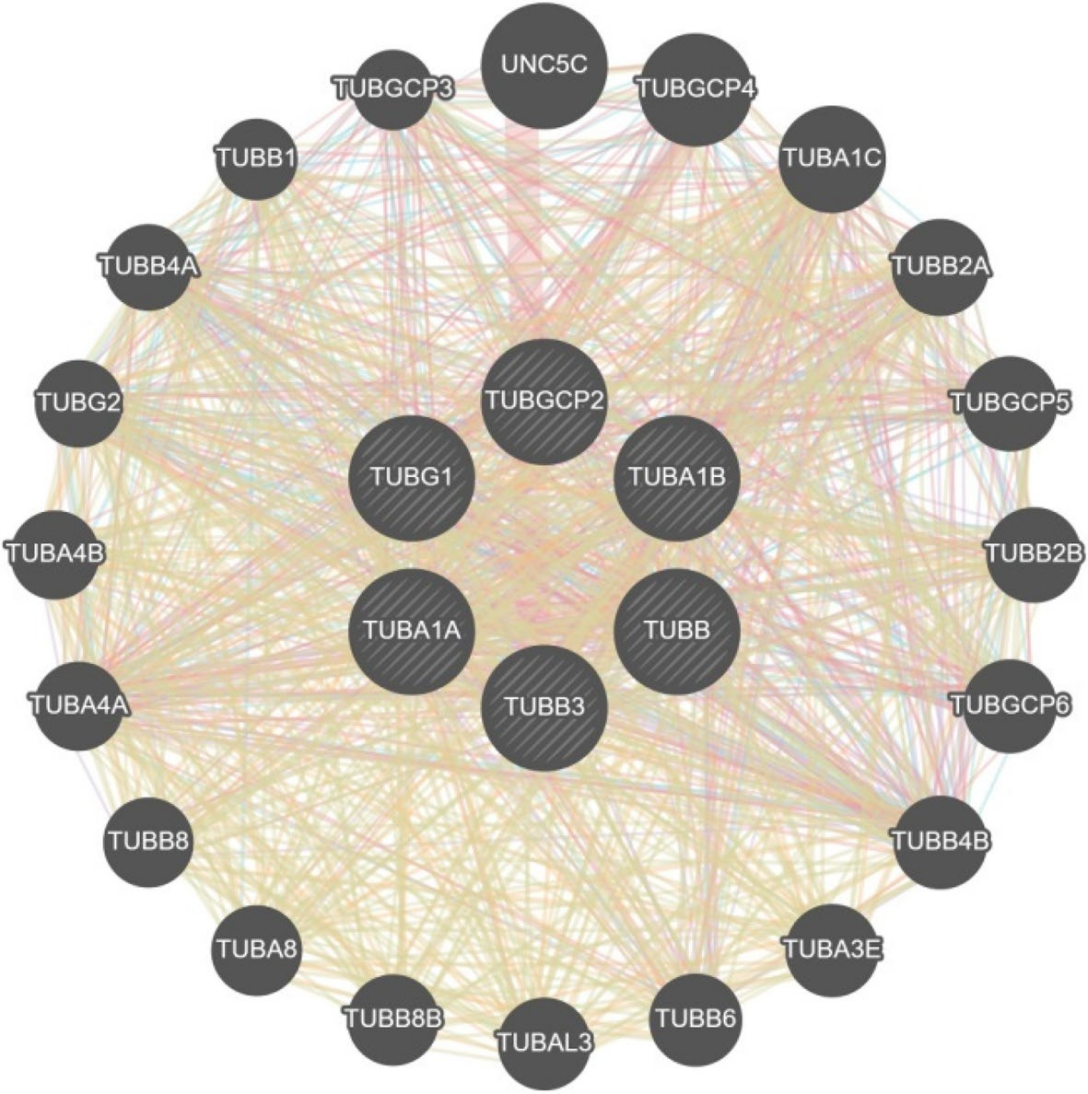
Six selected tubulin isoforms PPI in GeneMANIA. Totally, 1183 edges were detected between these 6 isoforms. Showing 20 related genes, with 26 total genes, 1177 total links, and no attributes

**Fig. 4:**
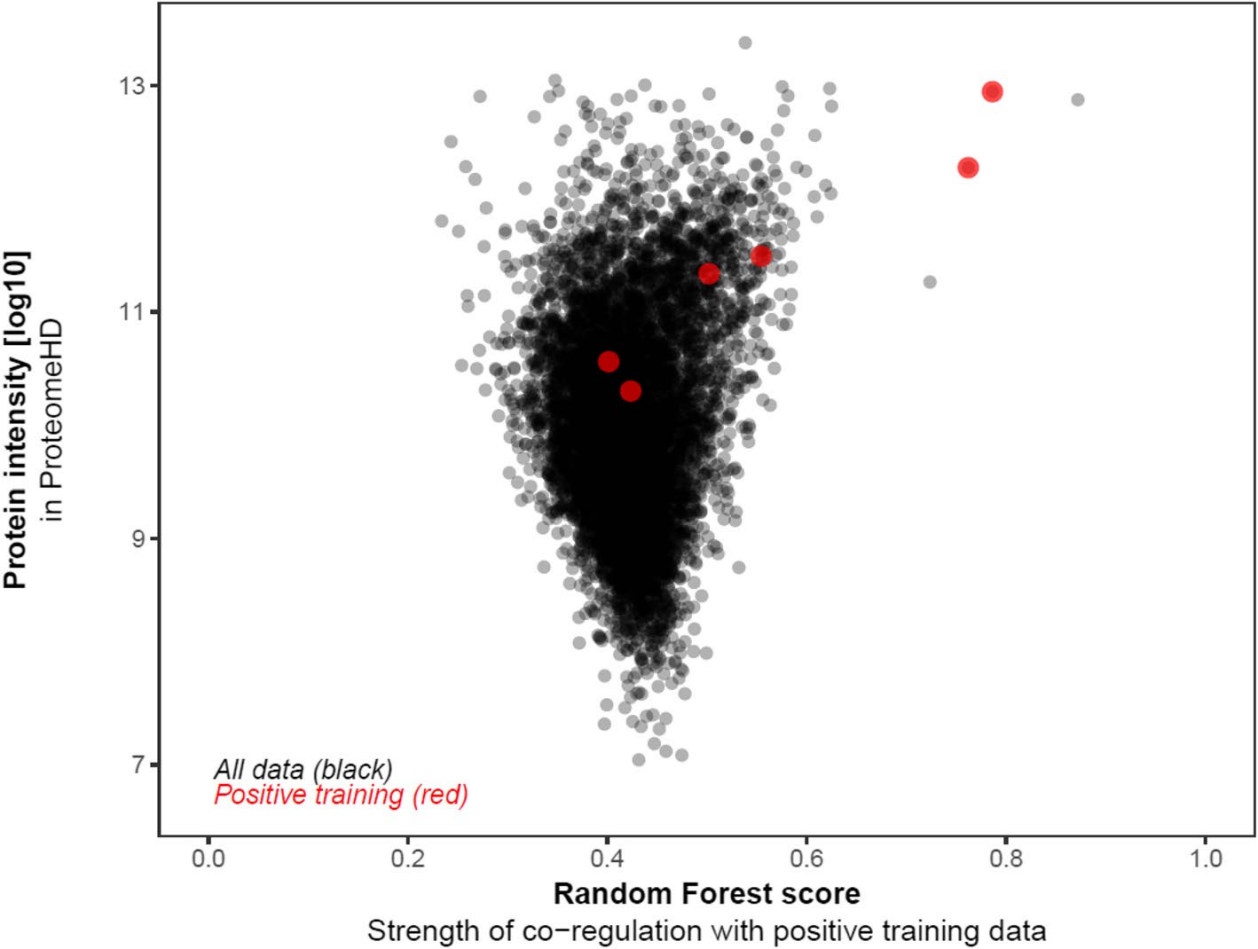
Six selected tubulin isoforms PPI in ProteomeHD. Cross-validated training data were used to assess performance, yielding an area under the precision-recall curve (AUPRC) of 0.379. (A random classifier would yield an AUPRC of 0.006.) Total protein interactions resulted identification of 7813 proteins

To identify tubulin-interacting proteins, the PPI analysis was carried out as described in flow chart (Fig. 1). In the first step, I used all 6TI together for PPI analysis in Stringbase, ProteomeHD, and GeneMANIA. In Stringbase, I observed 11 edges in PPIs between the score of 1 and 0.97 and observed all PPIs within tubulin family of isoforms (Fig. 2), GeneMANIA indicated 1183 edges in PPIs between the score of 1 and 0.26 (Fig. 3), and ProteomeHD indicated 7813 PPIs between the score of 0.87 and 0.61 (Fig. 4). Overall. in top 10 PPIs at the first level of selection, I found TUBGCP4, TUBB2B, TUBG2, TUBGCP2, TUBGCP3, TUBB8, TUBA1C, and TUBGCP5 proteins as new tubulin family interacting proteins and UNC5C, PKM, CLIC1, GAPDH, RPL4, PCBP1, AND PRDX1 as non-tubulin family proteins (Table 1).

**Table 1:**
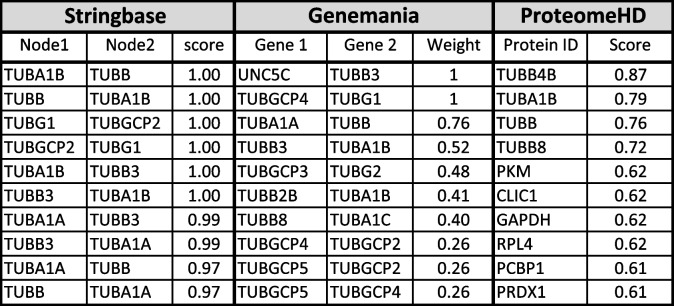
Using all 6 tubulin isoforms together in single search, top 10 PPI interactions in Stringbase, GeneMANIA, and ProteomeHD are shown with node-to-node interaction scores. As per ProteomeHD results format, single list column of interacting proteins is shown in last column

In the second level of PPI analysis, I used single tubulin isoform separately as key word using Stringbase, ProteomeHD, and GeneMANIA. In Stringbase, I identified 9 top-level interacting protein as functionally predicted partners with maximum number of 111 interacting proteins (Table 2). In ProteomeHD and GeneMANIA, top 10 interacting proteins were shown in Tables 3 and 4 with maximum number of 1193 and 852 PPIs, respectively. I pooled all top-level interacting proteins from the Table 1 and Tables 2, 3, and 4 resulting in identification of 224 tubulin and non-tubulin family of proteins altogether interacting directly with tubulin isoforms or indirectly associated with each isoform through co-regulation or co-expressions. For third level of PPI analysis, the pooled 224 proteins were used in GeneMANIA to identify more interacting protein, which resulted in identification of 4956 PPIs. Using this large dataset, I specifically selected top 10 proteins interacting with tubulin family based on weight of the edges (Table 5). In this third level of selection, I found TUBG1, TUBB3, TUBGCP4, TUBB, TUBA1B, TUBGCP6, AND TUBA1A as 7 top-level tubulin family of proteins strongly interacting with 6 other proteins such as HTT, DPYSL2, SKI, UNC5C, NINL, and DDX41, and I used these 13 proteins for high-level functional enrichment analysis. All the 6TI selected for PPI interactions showed strong node–node interaction data within themselves in all the databases. TUBGCP2 (*γ*-tubulin family) exhibited maximum number of PPIs among tubulin family. Though several non-tubulin families of protein interactions were observed as in Tables 2, 3, and 4, I selected HTT, DPYSL2, SKI, UNC5C, NINL, and DDX4 for functional enrichment analysis as they were found to be repeated in PPIs.

**Table 2:**
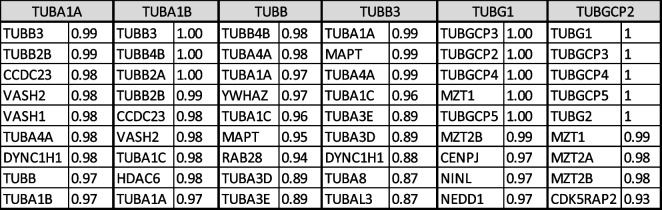
Stringbase PPI search was used with single tubulin isoform separately to identify functional predicted partners with highest interaction score. The total number of protein interactions varied between 71 and 111 node-to-node interactions

**Table 3:**
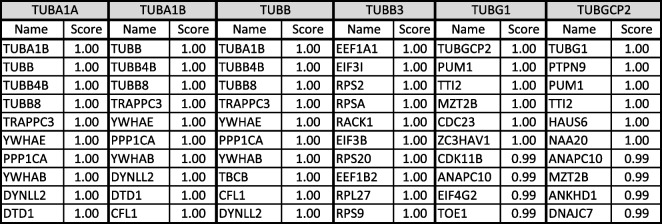
ProteomeHD PPI search was used with single tubulin isoform separately to identify functional predicted partners with highest interaction score. The total number of protein interactions varied between 157 and 1193 node-to-node interactions

**Table 4:**
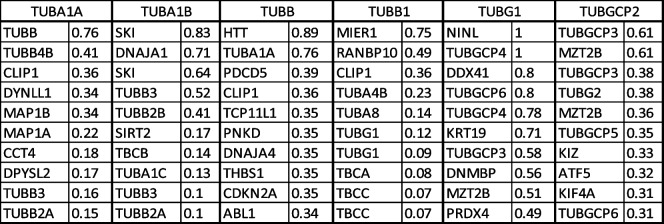
GeneMANIA search was used with single tubulin isoform separately to identify functional predicted partners with highest interaction score. The total number of protein interactions varied between 400 and 852 node-to-node interactions

**Table 5:**
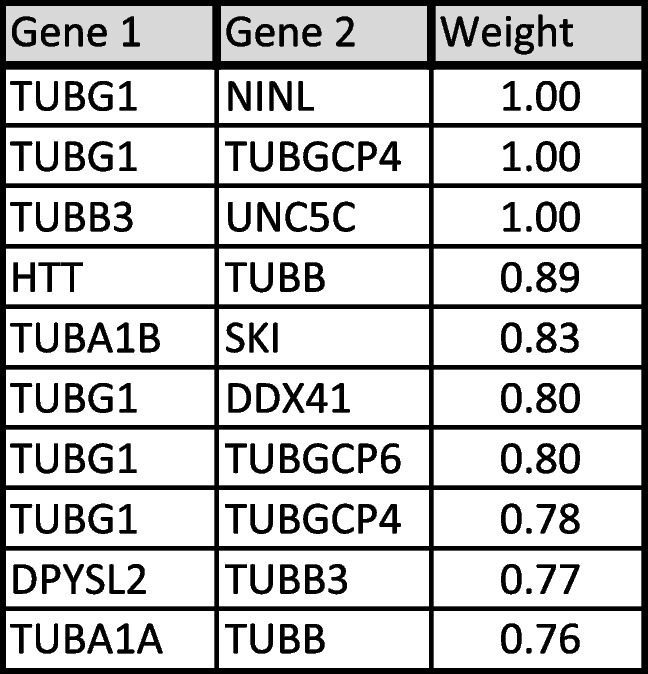
The pooled dataset of 224 genes from all the PPI databases was used in GeneMANIA to identify top ten strongly interacting proteins as shown in above table. The interactive values between two genes gene 1 and gene 2 are shown as weight. GeneMANIA search resulted in 4956 interacting nodal edges, and 13 proteins were identified from the table. The maximum interactions of *γ*-tubulin family (TUBG1, TUBGCP4, and TUBGCP6) were observed

### Functional enrichment analysis of strongly interacting proteins of tubulins

**Fig. 5:**
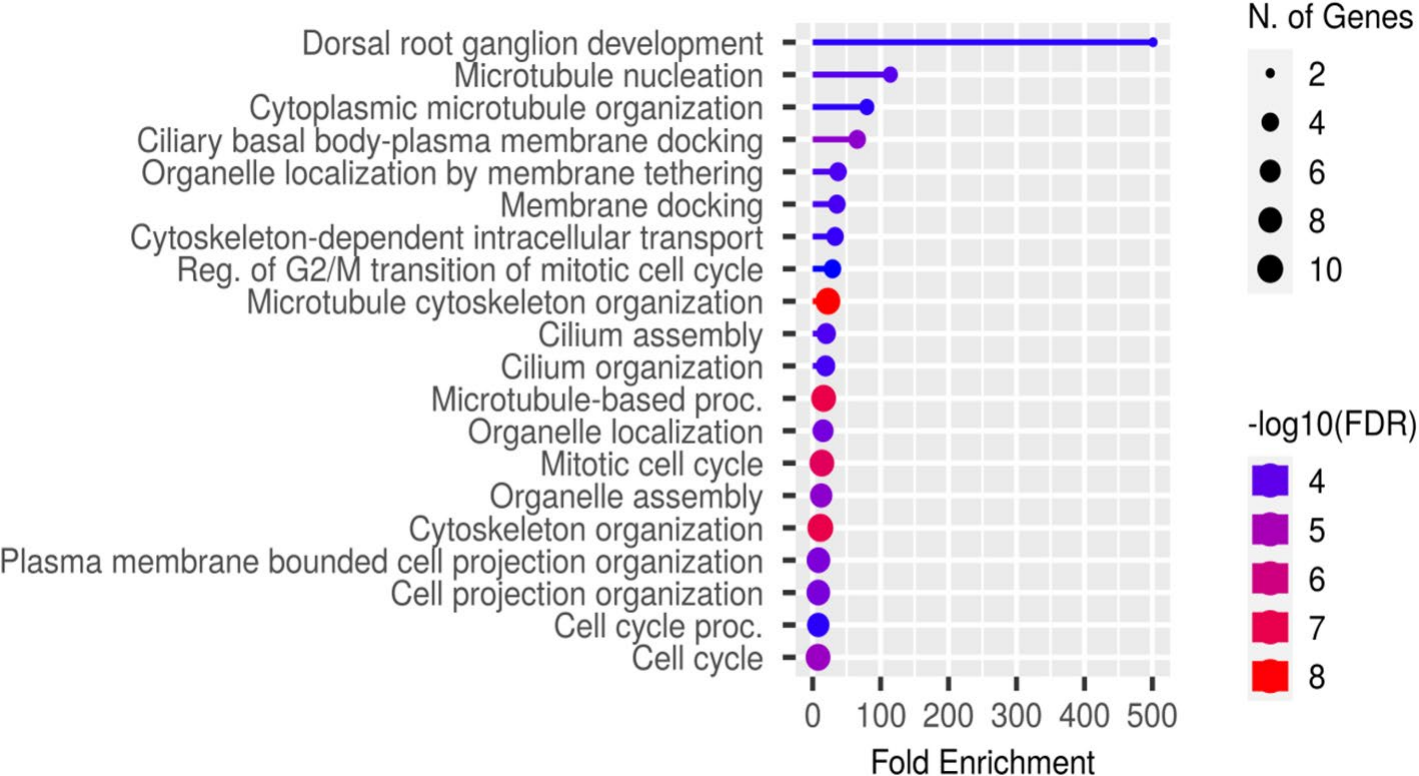
High-level functional enrichment analysis of top 13 strongly interacting proteins of tubulin in ShinyGO ontology

In first step of functional enrichment analysis, I determined the functional characteristics of 6TI together in GeneMANIA and observed a maximum of 15 genes in network associated with important functions in protein binding and localization of nucleic acids and chromosomes (Table 6). The 13 strongly interacting protein of tubulins exhibited similar functions as with 6TI, with maximum of 11 genes from the network and exhibited additional functions as microtubule binding, polymerization or depolymerization of microtubules, membrane tethering, membrane docking, cell cycle association functions, and enzyme activities (Table 7). Furthermore, high-level functional enrichment analysis using ShinyGO suggested microtubule and cell cycle functions with more than 100-fold functional enrichment in dorsal root ganglion development and microtubule nucleation (Fig. 5).

**Table 6:**
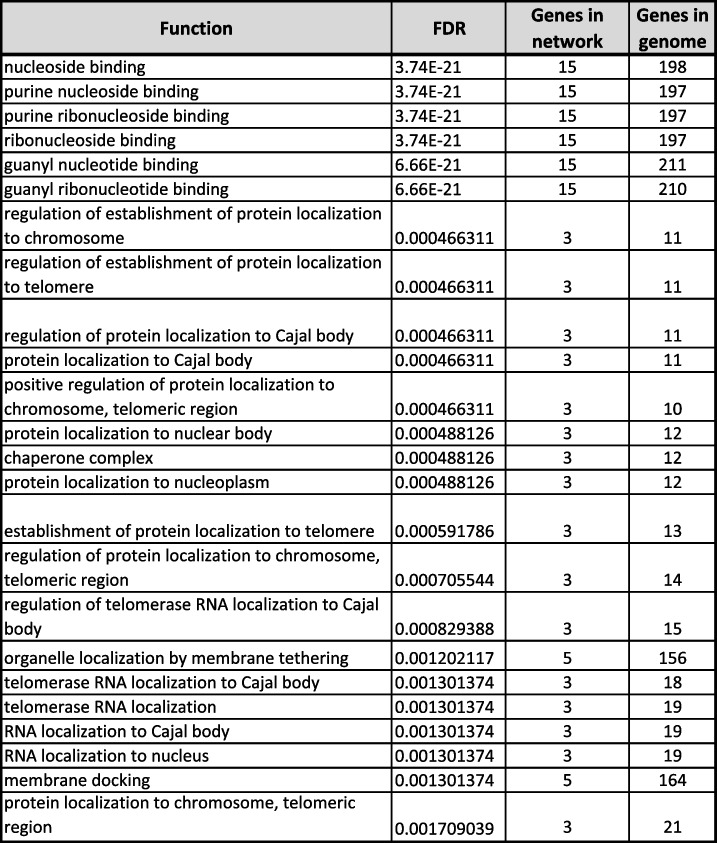
Functional enrichment analysis of six tubulin isoforms together in GeneMANIA. The functional significance of tubulins is listed starting with lowest FDR (false discovery rate) value

**Table 7:**
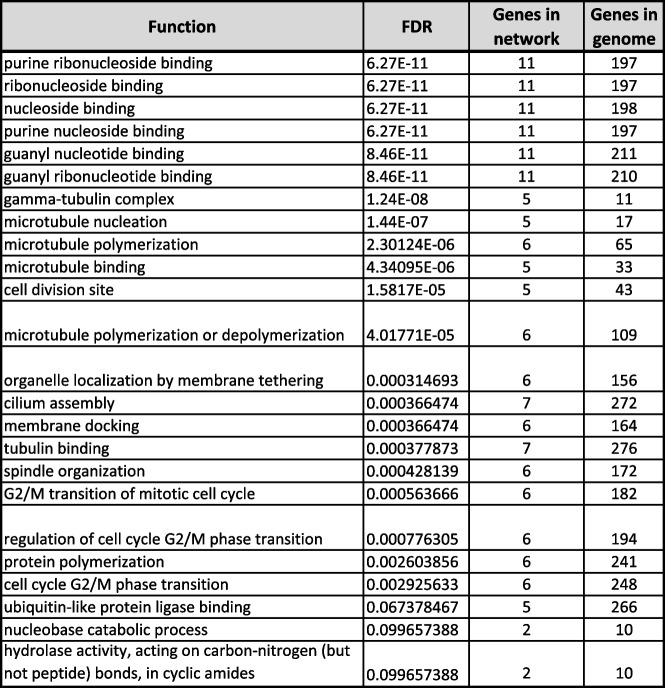
Functional enrichment analysis of top 13 strongly interacting proteins of tubulins in GeneMANIA. The functional significance of tubulins is listed starting with lowest FDR (false discovery rate) value

**Fig. 6:**
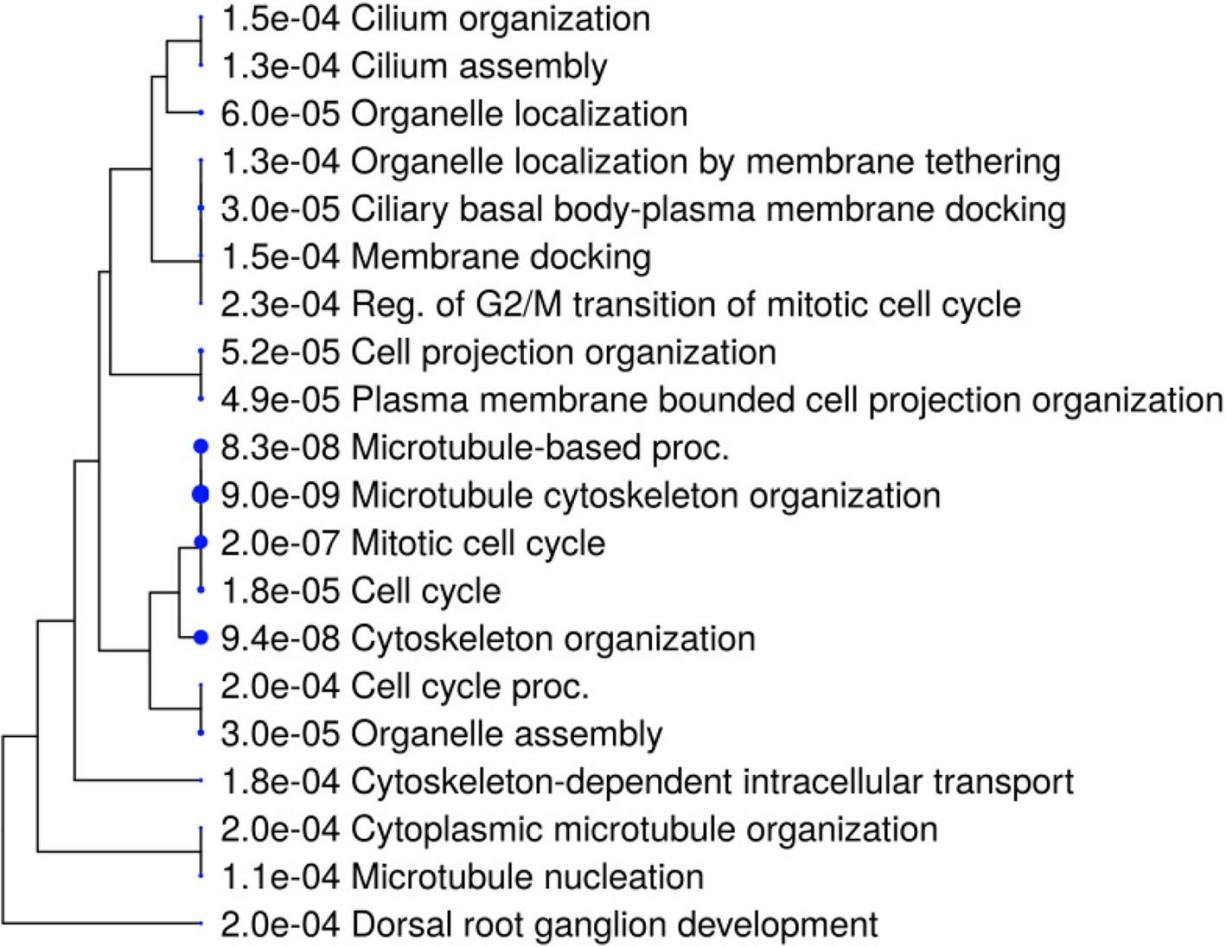
A hierarchical clustering tree summarizing the correlation among significant pathways of top 10 tubulin interacting genes in ShinyGO. Pathways with many shared genes were clustered together. Bigger dots indicate more significant *P*-values

To identify responsible genes contributing to specific function, I categorized the top 13 strongly interacting proteins of tubulin using ShinyGO. Out of 13 genes, maximum of 7 genes were shown to involve in cell cycle process, cellular component, and cellular localization. Additional functions were described as in Table 8. The results in Tables 5 and Table 8 indicated a maximum number repeated interactions of *γ*-tubulins family of proteins (TUBG1, TUBGCP4, & TUBBGCP6), which were known to contribute majorly for tubulin-associated functions compared with *α*-tubulin (TUBA1A) and *β*-tubulins (TUBB AND TUBB3). HTT, DPYSL2, SKI, UNC5C, NINL, and DDX were found closely associated with many of the tubulin-associated functions. I next evaluated correlation among significant pathways using tree dendrogram (Fig. 6), in which many shared genes were clustered together and observed highest significant values with microtubule organization, mitotic cell cycle, and cytoskeleton organization.

**Table 8:**
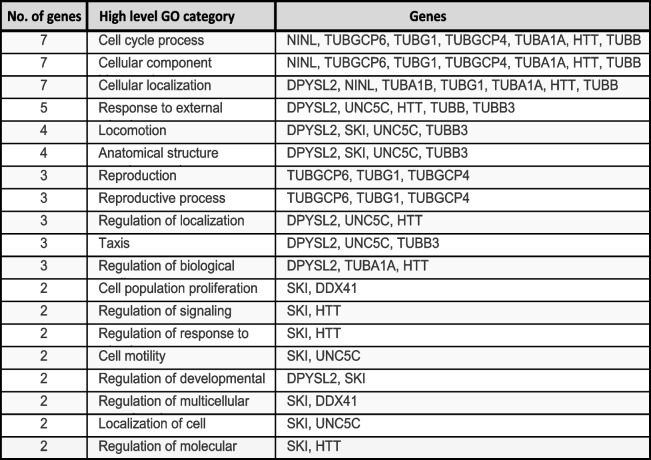
Groupings of high-level functional enrichment of top 13 strongly interacting tubulin proteins using ShinyGo. The maximum interactions of *γ*-tubulin family (TUBG1, TUBGCP4, and TUBGCP6) were found more

## Discussion

Increasing number of regulatory proteins and subpopulation of tubulin proteins are being reported with poor understanding in their association with microtubule assembly and disassembly. In different cell types, tubulin isotypes are expressed at different levels with various cellular functions by multiple genes [1, 2, 7, 23]. In this paper, the selection of 6TI was based on the manual curation, giving importance to all three tubulin classes (*α*-, *β*-, & *γ*-tubulins). Extensive literature search indicated high diversity of tubulin isotypes in various animal species, and in the present study, I selected two isotypes from each major class of *α*-, *β*-, and - γ - tubulins belonging to humans. All the 6TI previously exhibited important molecular functions in MT dynamics, and their functional abnormalities were reported in human disorders such as neuronal abnormalities, impaired motor behaviors, and cancers [24–29].

In our PPI analysis using 6TI, I shortlisted top level and strongly interacting proteins of tubulins out of several thousands of PPIs observed in GO databases. As described in methodology, these PPI data were obtained from curated GO databases. The majority of the results of tubulin PPIs were based on experimental findings from molecular interactions studies of tubulin isotypes in relation to MT dynamics through various signaling pathways, enzyme activity, gene fusions, mRNA co-expressions, co-regulations, or physical interactions [18–22].

In our initial PPI analysis, GeneMANIA identified 20 additional tubulin isotypes and a non-tubular protein UNC5C exhibiting strong interaction with TUBB3. Earlier studies reported that UNC5C (netrin receptor) directly interacted with TUBB3 in axon outgrowth of primary neurons and mediated microtubule polymerization which could be inhibited by its ligand netrin-1 in cosedimentation assay [30]. In initial round of PPI search, I found UNC5C (netrin receptor) interaction with TUBB3 as strong PPI interactions, which is a non-tubulin family of protein involved in neuronal development and also associated with Alzheimer’s disease [31].

Mutations in TUBB3 were previously reported in facial palsy and peripheral neuropathy [32] and also reported in the development of gallbladder cancers through Akt/mTOR signal pathway [27]. Other than 6TI, repeated PPIs of *γ*-tubulin subtypes (TUBGCP2, TUBGCP3, TUBGCP4, and TUBGCP5) were observed, and such involvement of *γ*-tubulins subtypes was previously reported to play essential role in GCP formation during assembly of microtubules. GCP abnormalities were earlier reported in cancer progression, retinopathy, and neuronal abnormalities [23, 33, 34]. Similarly, other additional tubulin isotypes TUBB2B, TUBB8, and TUBA1C, which I observed in first level of our selection steps, were previously reported to involve in nucleation of MT, cancer progression, oocyte maturation, and neuronal abnormalities [23, 35, 36].

Similarly, initial PPI analysis using 6TI together in Stringbase and GeneMANIA indicated strong PPIs of TUBGCP2 with other *γ*-tubulin subtypes (TUBG1, TUBGCP2, &TUBGCP4) and in the second round of PPI analysis of TUBGCP2 in all databases indicated its strong interactions with other *γ*-tubulin subtypes (TUBG1 AND TUBG2) as well as with several other GCP subtypes (TUBGCP3, TUBGCP4, TUBGCP5, and TUBGCP6) possibly indicating their combined functional role of these GCP subtypes in the formation of *γ*-tubulin ring complex (*γ*-TuRC). In third round of PPI analysis in GeneMANIA, I found several *γ*-tubulin subtypes interacting with non-tubulin family of proteins. The involvement of *γ*-tubulin in the formation of *γ*-TuRC was previously reported [3, 5, 37], and in the present study, I identified the involvement of additional *γ*-tubulin subtypes in the formation of *γ*-TuRC. In our three selection steps of PPI analysis using different databases, I found the interaction of TUBGCP2 with more number of *γ*-tubulin subtypes (TUBGCP3, TUBGCP4, TUBGCP5, and TUBGCP6). The possible role of *γ*-tubulin subtypes was earlier reported to regulate the nucleation of *α*-/*β*-tubulin heterodimers in microtubule assembly [37, 38]. In in vitro cellular assay, *γ*-tubulin was reported to form a complex with proliferating cell nuclear antigen (PCNA), and a significant correlation in expression of *γ*-tubulin (TUBG1) with PCNA was reported in tumor cells [39]. The association of non-tubulin family of proteins (MAPs) such as ChK2, C53, ATR, p53, BRCA1, and Rad51 with *γ*-tubulin isotypes was previously shown to regulate cell cycle and microtubule nucleation, which together act as a signal transduction hub in their functional network [40–43].

I found additional new tubulin family of isoforms and several other non-tubular family of proteins. TUBGCP2 (*γ*-tubulin) exhibited highest number of PPIs in selection steps, and other *γ*-tubulin family of proteins TUBG1, TUBGCP4, and TUBGCP6 was found in the final set of GeneMANIA as well as in high-level functional enrichment analysis in ShinyGO, possibly suggesting a significant functional role of *γ*-tubulins in microtubular processes as well as in the formation of *γ*-tubulin ring complex formation as described in literatures [5, 33, 34].

In initial round of selection process with 6TI together, I found TUBA1B as another top 7 tubulin family of protein interacting strongly with non-tubulin family of proteins; TUBA1B was also found strongly interacting with *β*-tubulin isotypes (TUBB and TUBB3) in Stringbase and GeneMANIA databases. TUBA1B was earlier found essential in the processes of cell cycle, spliceosome, and DNA replication [44, 45]. In the second round in different databases, TUBA1B strongly interacted with more number of *β*-tubulin isotypes compared to other isotypes possibly indicating its combined functional involvement with a few additional *β*-tubulin isotypes in MT dynamics.

The association of tubulin isotypes with non-tubulin proteins was largely dependent on various signaling pathways and posttranslational modifications of tubulins such as acetylation, phosphorylation, tyrosination, polyglutamylation, and methylation [27, 37, 46, 47]. The acetylation and phosphorylation of β-tubulin in reducing microtubule assembly were previously reported, and enzymes like minikinase/DYRK1a or cyclin-dependent kinase Cdk1 were involved in such processes [48]. Tyrosination was shown to regulate the recruitment of Clip-170 (a microtubule plus-end-tracking protein) to microtubule tips, and in addition, it was reported to recruit the motor protein dynein through its regulator dynactin which interacted directly with the *α*-tubulin tail [49–51]. SRC kinase-mediated tyrosine phosphorylation of TUBB3 was demonstrated to regulate mitotic spindle dynamics in prostate cancers [52]. In cancer stem cells, GLUT1, GRP78, VDAC, and Ephrins interacted with *β*-tubulin isotypes (e.g., βIVb) in maintaining cancer stem cell niches [53].

The uncontrollable growth in MT was earlier demonstrated with mutant TUBA1A, which exhibited weaker interaction with MAP protein such as XMAP215 [54]. In neural stem cell differentiation, the physical interaction of β3-tubulin (TUBB3) with dihydropyrimidinase-like 2 DPYSL2 (a family of collapsin response mediator) and Numb (neuronal protein) was demonstrated using GeneMANIA [55]. Disruption of *α*-tubulin 4a polyglutamylation prevents aggregation of hyper-phosphorylated tau (MAP protein) and microglia activation in mice [56].

In initial non-tubular protein interactions, I found PKM2, CLIC1, GAPDH, RPL4, PCB1, and PRDX1 in top six PPIs. Tumor-specific pyruvate kinase (PKM2) and chloride intracellular channel protein 1 (CLIC1) were earlier shown to involve in the process of cytokinesis and cell cycle progression, respectively [57, 58]. GAPDH was found as a known gene commonly used as housekeeping gene [59]. RPL4, PCB1, and PRDX1 were reported to involve in protein synthesis, maintenance of nucleolar structure, and antioxidant activities [60–62]. Based on our PPI results, I predicted the strong involvement of these non-tubular proteins functioning in association with our selected 6TI in MT dynamics.

Using ShinyGO, I was able to group the functional association of non-tubular proteins like HTT, DPYSL2, SKI, UNC5C, NINL, and DDX41 with tubulin isoforms. In GeneMANIA functional annotation studies, we found the involvement of all tubulin interacting proteins in nucleotide-binding, structural constituent of cytoskeleton, microtubule nucleation, microtubule polymerization or depolymerization, etc. I identified 13 strongly interacting proteins of tubulin based on highest node-to-node interaction score or weight of the interacting edges in PPI interactions. In order to refine and identify strongly interacting tubulin proteins, I repeated tubulin PPI search two times with all three web-based tools and finally with GeneMANIA for functional enrichment analysis. The high-level gene functional enrichment analysis using ShinyGO strongly suggested the significant functional involvement of 13 selected proteins in cell cycle process, microtubule assembly or disassembly, cellular component, and cellular localization. Based on grouping of top-level functional protein interactions, I found isoforms of *γ*-tubulins playing a major functional role which may be targeted as possible drug target. I found HTT (Huntington disease-causing gene) and UNC5C (netrin receptor) as top-level non-tubulin family of 6TI protein interactors; both were previously reported to involve in neurodevelopment by interacting with *β*-tubulin and tau proteins [30, 31, 63]. DPYSL2 (dihydropyrimidinase-like 2) and SKI (sphingosine kinase) were found essential in the assembly and stabilization of MT in various cell types and in cancers [55, 64]. NINL was earlier shown to control *γ*-tubules in stimulating MT nucleation [65], and DDX41 was shown to regulate RNA secondary structures [66].

In our combined output results from three different GO database, I found several additional tubulin isotypes and six additional non-tubular proteins HTT, DPYSL2, SKI, UNC5C, NINL, AND DDX4 repeatedly in PPIs. They were selected for functional enrichment analysis in GeneMANIA and ShinyGO, since these two databases were able to significantly group the functional genes in user-friendly downloadable format. Taking advantage of these GO database, I was able to shortlist the top-level tubulin functional partners based on their aggregated functional roles, significance of FDR values, and significant *P*-values, correlating significant pathways and number of folds in functional enrichments. In our studies, I was able to narrow down the top-level tubulin protein interactors from several thousands of PPIs results observed in databases. Our approach of identifying top-level tubulin family of proteins and their associated non-tubulin family of functional partners yielded several insights in prioritizing tubulins as drug targets.

Tubulin has gained attention as a fundamental target in anticancer therapeutic approaches since they play an essential role in cell division [27, 34, 44, 47, 48], and several microtubule-targeting agents (MTA) have been employed as anticancer drugs [67, 68]. In cancer cells, the interaction of MT with motor proteins like dynein, kinesin, and myosin was reported as crucial protein mediators in cell proliferation and invasion [69]. Therefore, the identification of MT signaling pathways through PPI studies may offer a source of novel anticancer treatments through identification of essential genes having more number of PPIs in network as HUB genes with positive feedback function [70, 71]. A few studies constructed a PPI network using cBioPortal, STRING, and KEGG pathway analysis using 50 frequently altered MAP genes and highlighted their potential application in cancer treatment and prognosis [72, 73].

## Conclusion

This research identified the top-level tubulin family of proteins and its functional partners based on their multiple functional involvements. To improve the data coverage, I used three different user-friendly web-based GO databases and refined the selection process of tubulins by repeating PPI analysis three times. Gene Ontology and protein network-based interactive analysis are increasingly gaining importance in elucidating the functions of novel and druggable genes. Considering the complexity in various GO data resources, developing a simplified computational, machine learning, and genetic algorithms approaches will be highly beneficial for understanding disease gene relationship and therapeutic purposes.

## Data Availability

All data analyzed during this study are included in this article.

## Abbreviations

PPIs: Protein-protein interactions
GO: Gene Ontology
MAP: Microtubule-associated proteins
GTP: Guanine triphosphate
VDAC: Tubulin in voltage-dependent anion channel
6TI: Six tubulin isoforms
AUPRC: Area under the precision recall curve
FDR: False discovery rate
